# Mitigating Disparity in Health-care Resources Between Countries for Management of Hereditary Angioedema

**DOI:** 10.1007/s12016-021-08854-5

**Published:** 2021-05-18

**Authors:** Ankur Kumar Jindal, Avner Reshef, Hilary Longhurst, GEHM workgroup (Global Equity in HAE Management), Werner Aberer, Stephen Betchel, Konrad Bork, Emel Aygören-Pürsün, Marcus Maurer, Markus Magerl, Laurence Bouillet, Anette Bygum, Teresa Caballero, Mauro Cancian, Henriette Farkas, Vesna Grivcheva-Panovska, Anete Grumach, Okan Gulbahar, Michihiro Hide, Ankur Jindal, Surjit Singh, Hye-Ryun Kang, Avner Reshef, Aharon Kessel, Hilary Longhurst, Karen Lindsay, Anthony Jordan, Rohan Ameratunga, William Lumry, Jonathan Bernstein, Timothy Craig, Marc Riedl, Don Levy, Alejandro Malbran, Anastasios Germenis, Fotis Psarros, Marcin Stobiecki, Grzegorz Porebski, Anna Valerieva, Fiona Wardman, Youjia Zhong, Christina Weber

**Affiliations:** 1 Allergy Immunology Unit, Department of Paediatrics, Advanced Paediatrics Centre, Postgraduate Institute of Medical Education and Research, Chandigarh, India; 2 Allergy, Immunology & Angioedema Center, Barzilai University Medical Center, Ashkelon, Israel; 3 Senior Medical Officer, Auckland District Health Board, Auckland, New Zealand; 4 Department of Allergy and Immunology, University College Hospitals, London, England; 5 Department of Medicine, University of Auckland, Auckland, New Zealand

**Keywords:** Angioedema, Clinical guidelines, Disparity, Inequity, Medications, Treatment

## Abstract

Hereditary angioedema (HAE) is a rare genetic disorder characterized by recurrent episodes of skin and mucosal edema. The main treatment goal is to enable a “normal life” for all patients. However, due to high costs, there are limited options for the management of HAE in most developing and low-income countries. As a result, most of the recommended first-line treatments are not available. In this review, we attempt to highlight the disparities in health-care resources for the management of patients with HAE amongst different countries. Data was collected from HAE experts in countries who provide tabulated information regarding management and availability of HAE treatments in their countries. We reviewed the two most recent international HAE guidelines. Using India, the world’s second most populous country, as a paradigm for HAE management in lower-income countries, we reviewed the evidence for second-line and non-recommended practices reported by HAE experts. Results suggest significant inequities in provision of HAE services and treatments. HAE patients in low-income countries do not have access to life-saving acute drugs or recently developed highly effective prophylactic medications. Most low-income countries do not have specialized HAE services or diagnostic facilities, resulting in consequent long delays in diagnosis. Suggestions for optimizing the use of limited resources as a basis for future discussion and reaching a global consensus are provided. There is an urgent need to improve HAE services, diagnostics and treatments currently available to lower-income countries. We recommend that all HAE stakeholders support the need for global equity and access to these essential measures.

**Supplementary Information:**

The online version contains supplementary material available at 10.1007/s12016-021-08854-5.

## Introduction

Hereditary angioedema (HAE) is an uncommon primary immunodeficiency disease characterized clinically by recurrent episodes of skin and mucosal edema. C1-INH deficiency leading to dysregulated bradykinin production is the most common cause. HAE is a chronic disorder and may occasionally lead to mortality due to laryngeal edema. Mortality in patients with HAE (caused by laryngeal edema) was approximately 30% in the past [1]. However, with the availability and access to better treatment modalities for HAE, mortality has been significantly reduced, at least in high-income countries [2, 3]. C1-INH replacement therapy given subcutaneously or intravenously as long-term prophylaxis or intravenously to treat an acute episode is a highly effective and commonly used therapy [4, 5]. In addition, several other medications have been approved and are recommended for management of patients with HAE [4, 5]. These include icatibant and ecallantide for acute attacks and lanadelumab for prophylaxis [6]. Because of the availability and access to these medications in most developed countries, mortality due to HAE has been significantly reduced [7]. However, the mortality in undiagnosed patients has been found to be much higher as compared with diagnosed patients [7]. As there is as yet no permanent cure for this disease, patients continue to experience impaired quality of life. However, quality of life and socioeconomic function appear to be improving thanks to better access to modern treatments [8].

In contrast, there are limited options for the management of HAE in most developing and low-income countries and often none of the first-line treatments are available. Epidemiological data on HAE is also not available from these countries. Patients with HAE in these countries are frequently managed prophylactically using attenuated androgens or tranexamic acid and for short-term prophylaxis and acute “on-demand” therapy with attenuated androgens and fresh frozen plasma (FFP), respectively. Thus, in these countries, it is not uncommon to see avoidable HAE-related mortality and increased morbidity [9].

In this review, we attempt to highlight the disparities in health-care resources for management of patients with HAE amongst different countries. We also suggest pragmatic solutions for improving care in lower-income countries where access to first-line treatments are currently not available.

## Methods

### Data Collection From Individual Countries

**Fig. 1:**
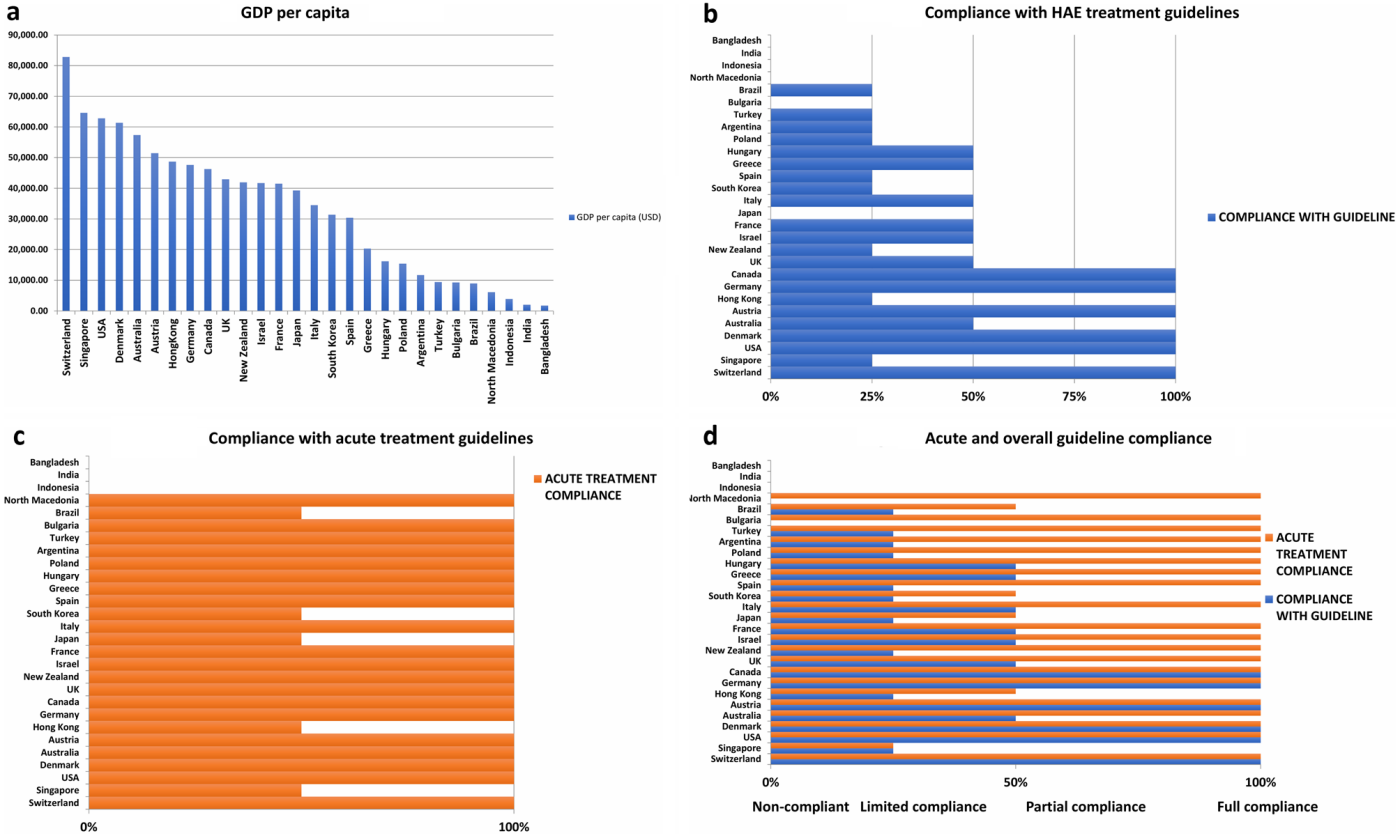
**a**: GDP per capita (2018). **b** Compliance with acute treatment guideline. **c** Compliance with international treatment guideline. **d** Acute and overall international treatment guideline compliance

Thirty-six HAE experts from 28 countries were contacted via email and requested to provide information regarding the management and availability of HAE treatments in their countries (Table 2). Countries were ranked according to GDP per capita 2018 [10] (Fig. 1a) and assessed for compliance with international HAE guidelines. Full compliance to international guidelines was assigned to countries where all HAE patients had access to first-line acute (on-demand) treatment (plasma or recombinant intravenous C1-INH, icatibant or ecallantide) that could be self-administered at home by the patient or under the care of a health-care provider and, when appropriate, to first-line approved prophylactic treatments (IV or subcutaneous C1-INH or lanadelumab). Partial compliance was assigned when first-line acute treatment was readily available but where home therapy was not routine, or where there were restrictions on access to first-line prophylactic medications. Non-compliance was assigned if all first-line acute treatments were not readily available or when prophylactic treatment was not available.

### Review of Published Guidelines

The two most recent international HAE guidelines were reviewed [4, 5]. We gauged management of HAE in the countries who responded against these guidelines, grading adherence to the guidelines as ‘compliant’ (where patients with HAE were able to be treated according to guideline, including lanadelumab or subcutaneous C1-INH for prophylaxis), ‘partial compliance’ (where there were limitations in access to recommended care, according to patient attack frequency or other characteristics) or ‘non-compliance’ where patients did not routinely have access to the recommended first-line care. Full compliance was assigned to countries where all HAE patients had access to first-line acute treatment (intravenous C1-INH, icatibant or ecallantide) administered at home and, where required, to first-line prophylactic treatments (subcutaneous/intravenous C1-INH or lanadelumab). Partial compliance was assigned when first-line acute treatment was readily available but where home therapy was not routine, or where there were restrictions on access to first-line prophylactic medications. Limited compliance was assigned where first-line acute treatments were available but where first-line prophylactic medications were not available. Non-compliance was assigned if all first-line acute treatments were not readily available or when prophylactic treatment was not available. We also measured access to acute care facilities according to the same criteria. Compliance is arbitrarily assigned to 100%, partial compliance 50% and limited compliance 25% in the figures.

Using India, the world’s second most populous country and its biggest democracy, as a paradigm for HAE management in lower-income countries, we reviewed and compared with countries in compliance with HAE guidelines the evidence for second-line and non-recommended practices reported by HAE experts.

We additionally provide suggestions aimed at generating worldwide support for local access to current and future first-line therapies to ensure safe, effective HAE therapy for all of those affected, regardless of national healthcare economics.

## Results

HAE experts from 28 countries provided information about nationwide availability of HAE treatments electronically. The HAE International patient association provided data for four countries (Tables 1 and 2). Only six of the 28 countries, all with GDP/capita of over US$40,000, had the resources to fully comply with international guidelines. An additional 16 countries had limited or partial compliance mostly due to unavailability or limitations placed on the use of modern highly effective but costly prophylactic agents. Six countries, of which five were low income-GDP/capita of less than $10,000-were non-compliant (Fig. 1a-d ).

**Table 1:** List of the Global Equity in HAE Management (GEHM) workgroup participants

	Country	Name		Affiliation	Mail
1	Austria	Aberer	Werner	Medical University of Graz, Graz, Austria	werner.aberer@medunigraz.at
2	Canada	Betchel	Stephen	Univ. of Toronto, St. Michael's Hospital, Toronto, Canada	Stephen.Betschel@unityhealth.to
3	Germany	Bork Aygören-Pürsün Maurer Magerl	Konrad Emel Marcus Markus	Universitäts-Hautklinik, Dermatology, Mainz, Germany Goethe-Universität, Frankfurt am Main, Germany Charité - Universitätsmedizin Berlin, Berlin, Germany Charité - Universitätsmedizin Berlin, Berlin, Germany	konrad.bork@unimedizin-mainz.de aygoeren@em.uni-frankfurt.de marcus.maurer@charite.de markus.magerl@charite.de
4	France	Bouillet	Laurence	Chercheur à Université Grenoble Alpes, Grenoble, France	LBouillet@chu-grenoble.fr
5	Denmark	Bygum	Anette	Odense Universitetshospital, Odense, Denmark	Anette.Bygum@rsyd.dk
6	Spain	Caballero	Teresa	Hospital Universitario La Paz, Madrid, Spain	tercaballero@gmail.com
7	Italy	Cancian	Mauro	University Hospital of Padova, Padova, Italy	mcancian@unipd.it
8	Hungary	Farkas	Henriette	Angioedema Ctr, Semmelweis University, Budapest, Hungary	farkas.henriette@med.semmelweis-univ.hu
9	North Macedonia	Grivcheva-Panovska	Vesna	University Sts Cyril and Methodius Skopje, North Macedonia	vesna_grivcheva_panovska@yahoo.com
10	Brazil	Grumach	Anete	Ctr for Rare Diseases, Faculdade de Medicina, São Paulo, Brazil	anete@grumach.com
11	Turkey	Gulbahar	Okan	Ege Üniversitesi, Izmir, Turkey	okan.gulbahar@yahoo.com
12	Japan	Hide	Michihiro	Dept of Dermatology, Hiroshima Univ. Hiroshima, Japan	ed1h-w1de-road@hiroshima-u.ac.jp
13	India	Jindal Singh	Ankur Surjit	Postgraduate Institute of Medical Education and Research, Chandigarh, India Postgraduate Institute of Medical Education and Research, Chandigarh, India	ankurjindal11@gmail.com surjitsinghpgi@rediffmail.com
14	Bangladesh	Jindal	Ankur	Postgraduate Institute of Medical Education and Research, Chandigarh, India	ankurjindal11@gmail.com
15	South Korea	Kang	Hye-Ryun	Seoul National University Hospital, Seoul, South Korea	helenmed@snu.ac.kr
16	Israel	Reshef Kessel	Avner Aharon	Barzilai University Medical Ctr, Ashkelon, Israel Bnay-Zion Med Ctr, Technion Medical School, Haifa, Israel	avnerre@bmc.gov.il aharon.kessel@b-zion.org.il
17	United Kingdom	Longhurst	Hilary	UCLH, London, UK Department of Medicine, University of Auckland, New Zealand	hlonghurst@doctors.org.uk
18	New Zealand	Lindsay Jordan Ameratunga	Karen Anthony Rohan	Auckland District Health Board Auckland District Health Board Department of Molecular Medicine and Pathology Faculty of Medical and Health Sciences, University of Auckland	KLindsay@adhb.govt.nz AnthonyJ@adhb.govt.nz rame001@aucklanduni.ac.nz
19	USA	Lumry Bernstein Craig Riedl Levy	William Jonathan Timothy Marc Don	Allergy & Immunology Assoc., Dallas TX, USA Univ. Cincinnati, Div. of Immunology, Cincinnati OH, USA Penn State University Hershey, PA, USA US HAEA Angioedema Ctr, Univ. of California, San Diego CA USA University of California at Irvine, Irvine, CA, USA	Lumrymd@me.com BERNSTJA@ucmail.uc.edu tcraig@pennstatehealth.psu.edu mriedl@health.ucsd.edu DLevy1@uci.edu
20	Argentina	Malbran	Alejandro	Asociación Argentina de AH, Buenos Aires, Argentina	amalbran31@hotmail.com
21	Greece	Germenis Psarros	Anastasios Fotis	School of Medicine, University of Thessaly, Larissa, Greece Greek Navy Hospital, Athens, Greece	agermen@med.uth.gr psarros@allergy.gr
22	Poland	Stobiecki Porebski	Marcin Grzegorz	Jagelonian University, Krakow, Poland Department of Clinical and Environmental Allergology, Jagiellonian University Medical College ul. Botaniczna 3, Krakow, Poland	marcin.stobiecki@uj.edu.pl g.porebski@uj.edu.pl
23	Bulgaria	Valerieva	Anna	University Hospital “Alexandrovska”, Sofia, Bulgaria	anna.valerieva@gmail.com
24 25 26	Australia Indonesia Hong Kong	Wardman	Fiona	HAE International (HAEi), Chief Regional Patient Advocate and Regional Patient Advocate, Asia Pacific	f.wardman@haei.org
27	Singapore	Wardman Zhong	Fiona Youjia	HAE International (HAEi), Chief Regional Patient Advocate and Regional Patient Advocate, Asia Pacific National University Hospital, Singapore	f.wardman@haei.org youjia_zhong@nuhs.edu.sg
28	Switzerland	Weber	Christina	Allergiestation, Universitätsspital Zürich, Switzerland	weber@chinderarztpraxis.ch

**Table 2:** HAE treatment by countries’ gross domestic product (GDP) per capital

Country	GDP/Cap ($)	Diagnosed with HAE (est.)	Guidelines	Acute treatment	Short-term prophylaxis	Long-term prophylaxis
Bangladesh	1,698	10	No	FFP	Dnz, FFP	Dnz, TA
India	2,010	130	No	FFP	Sta, Dnz, FFP	Dnz, Sta, TA
Indonesia	3,894	5	No	No	FFP	none
N. Macedonia	6,084	40	WAO/EAACI	pdC1-INH, rC1-INH	pdC1-INH, rC1-INH	not available
Brazil	8,921	1000	WAO/EAACI	Ict, pdC1-INH	pdC1-INH	Dnz, Ox, TA, FFP
Bulgaria	9,273	92	WAO/EAACI/ local	Ict, pdC1-INH, rC1-INH	rC1-INH	Not available
Turkey	9,370	700	WAO/EAACI	Ict, pdC1-INH, FFP	pdC1-INH	Dnz, TA, pdC1-INH
Argentina	11,684	500	Local	Ict, pdC1-INH	Dnz, TA, Ict, C1 INH	Dnz, TA, pdC1 INH
Poland	15,421	430	Local	Ict, pdC1-INH, rC1-INH	pdC1-INH	Dnz, TA, FFP
Hungary	16,162	198	WAO/EAACI/ local	Ict, pdC1-INH, rC1-INH	pdC1-INH, Dnz	Dnz, TA, pdC1-INH, Lan
Greece	20,324	179	WAO/EAACI	Ict, pdC1-INH	pdC1-INH, Dnz	Dnz, TA, Prog, pdC1-INH, Lan
Spain	30,371	1000	Local	Ict, pdC1-INH	pdC1-INH, Dnz	Dnz, Sta, TA, pdC1-INH
South Korea	31,363	70	No	Ict, FFP	Dnz	Dnz, TA
Italy	34,483	980	Local	Ict, pdC1-INH, rC1-INH	pdC1-INH, Dnz	Dnz, pdC1-INH, Lan
Japan	39,290	450	Local/WAO/EAACI	Ict, pdC1-INH	pdC1-INH	Danzol, TA
France	41,464	1500	WAO/EAACI	Ict, pdC1-INH, rC1-INH	pdC1-INH and rC1-INH	Dnz, TA, Prog, pdC1-INH, rC1-INH, Lan
Israel	41,715	300	Local	Ict, pdC1-INH, rC1-INH	pdC1-INH, rC1-INH	Dnz, TA, Lan
New Zealand	41,945	53	Local	Ict, pdC1-INH, rC1-INH	pdC1-INH, rC1-INH	Dnz, Sta, TA, pdC1-INH
UK	42,944	600	Local	Ict, pdC1-INH, rC1-INH	pdC1-INH, rC1-INH, Dnz, Ox	Dnz, Ox, TA, Prog, pdC1-INH*, Lan*
Canada	46,233	800	Canadian/Intl	pdC1-INH, Ict, FFP	pdC1-INH, Dnz	Dnz, pdC1-INH, Lan
Germany	47,603	1800	Local	Ict, pdC1-INH, rC1-INH	pdC1-INH, rC1-INH	Dnz, Ox, TA, pdC1-INH, Lan
Hong Kong	48,676	25	No	pdC1INH, FFP	FFP, pdC1-INH	Dnz, TA
Austria	51,462	120	WAO/EAACI	Ict, pdC1-INH,rC1-INH	pdC1-INH, rC1-INH, Dnz	Dnz, pdC1-INH, rC1-INH, Lan
Australia	57,374	270	ASCIA	Ict, pdC1-INH	Icat, pdC1-INH	pdC1-INH, TA
Denmark	61,350	112	WAO/EAACI	Ict, pdC1-INH, rC1-INH	pdC1-INH and rC1-INH	Dnz, TA, pdC1-INH, Lan
USA	62,795	6500	WAO/EAACI	Ict, Eca,pdC1-INH,rC1-INH	pdC1-INH, Dnz	Dnz, TA, pdC1-INH, Lan
Singapore	64,582	15	No	FFP, pdC1INH	FFP, pdC1-INH	Dnz, TA
Switzerland	82,797	130	WAO/EAACI	pdC1-INH	pdC1-INH	pdC1-INH, Dnz, Lan
Total No. of patients 17,879						

If availability of recommended acute treatments were considered as an isolated attribute of guideline adherence, then 20 countries were fully compliant, including most middle- and some lower-income countries whereas five countries were partially compliant mostly due to access limitations or requirement to be treated in a hospital and four low-income countries were non-compliant due to lack of C1-INH or icatibant in-patient or out-patient access (Fig. 1c). Figure 1d demonstrates that poor treatment guideline compliance in countries ranked according to GDP/capita is directly associated with a low GDP.

### Review of Guidelines and Suggestions for Optimal Use of Second-Line Agents

#### Diagnosis

All of the previously published HAE guidelines recommend that diagnosis of HAE with C1-INH deficiency (C1-INH-HAE) should be made by measuring C4, antigenic and functional C1-INH. Genetic diagnosis is not routinely required except in cases trying to differentiate de novo C1-INH mutations from acquired angioedema. The guidelines also recommend that all at risk relatives also be screened [4, 5].

The worldwide prevalence of HAE has been estimated to be approximately 1:50,000 [11]. However, there very little to no epidemiologic data from India and other less developed countries. Considering that the current population of India is approximately 1.38 billion, it is likely that 27,000 patients with HAE exist in the country. However, to the best of our knowledge, there are presently less than 200 diagnosed HAE patients in India. This suggests that 97.26% of patients with HAE remain undiagnosed. A similar situation is likely existing in other low-income countries in Africa and Southeast Asia. Thus, India illustrates the unfortunate and disparate situation seen for HAE patients in many low-income countries compared with higher-income countries. It has been reported that delays in diagnosis of HAE are very common in India, with a median delay in diagnosis of 6.5 years (range 0–28 years) [9]. Of note, it is very typical for parents or grandparents not to receive their initial diagnosis of HAE until their children or grandchildren begin to have symptoms [9]. There are several reasons for these shortfalls:

1. Lack of awareness about HAE amongst physicians and the lay public.
2. Very few trained immunologists in India. The Postgraduate Institute of Medical Education and Research (PGIMER) in Chandigarh, India, is the only medical centre in the country where a formal 3-year training program in Paediatric Immunology is provided. To date, 11 physicians have received training under this program and are now working in different parts of the country. In addition, there are a few centres in India that are providing training for Adult Immunology including the PGIMER in Chandigarh.
3. Diagnostic facilities for HAE, including genetic testing, are limited and mostly available only at tertiary-care referral hospitals.
4. Most hospitals in India would rely only on C4 and C1-INH quantitative levels to confirm or rule out a diagnosis of HAE. The assays for C1-INH quantitative levels are usually carried out in commercial labs using radial immunodiffusion or sometimes nephelometry. C1-INH function, which requires careful sample handling and a high level of technical expertise, is particularly challenging and not generally available [12] which may account for the lack of diagnosed patients with HAE, type 2.

Our experience with *SERPING1* gene sequencing in patients with type 1 HAE at PGIMER in Chandigarh (India) has shown 9 novel (likely pathogenic) and 2 previously reported disease-causing variants in 11 families where a SERPING1 variant could be detected. No disease-causing variants in SERPING1 gene were detected in 9 other suspected families tested [9]. Thus, in our practice, genetic diagnosis failed to diagnose 9/20 (diagnostic sensitivity of 45%) of those with biochemically proven quantitative C1-INH deficiency, while incautious interpretation of variants of unknown significance might risk false positives, reducing specificity of the test. This finding indicates the need to improve access to functional C1-INH assays and differentiate HAE from acquired angioedema which is caused by an anti-C1-INH antibody or an underlying paraproteinemia. Recently, real-time very stable dried blood-spot assays have been developed for functional C1-INH that may address the need for a reliable functional assay in developing countries like India [13]. However, genetic testing may be an effective strategy for diagnosis of family members where the disease-causing gene variant is known and where likely pathogenic variants can be detected. Affordable, genetic testing could overcome otherwise insurmountable challenges in sample transport and thus potentially become a gold standard for definitively diagnosing HAE. Additionally, if C4 and C1-INH are normal in a patient suspected to have HAE, at some centres no further testing is performed and these patients are treated as non-HAE angioedema. Thus, patients with HAE-nl-C1-INH may remain undiagnosed and untreated. In these cases, guidelines emphasize the importance of having heightened clinical suspicion of HAE, based on the presence of angioedema without wheals/urticaria, resistance to regular high dose, second-generation antihistamines and for those with a well-defined family history of angioedema and/or angioedema induced by estrogen. Genetic testing can address the small number of these families with previously defined Factor XII, plasminogen, kininogen and angiopoietin genetic mutations associated with normal complement HAE. However, similar to high-income countries, clinical diagnosis remains essential for the majority of normal complement HAE patients.

Other low-income countries are likely to face similar challenges and, in many countries, there are no immunologists or HAE specialists [HAE International (HAEi), personal communication]. Thus, wider physician recognition of HAE symptoms and clinical characteristics as well as access to pragmatic diagnostic pathways are urgently required in these countries.

We propose Box 1.

#### Box 1 Proposal for a Wider Physician Recognition of HAE Symptoms and Clinical Characteristics as Well as Access to Pragmatic Diagnostic Pathways

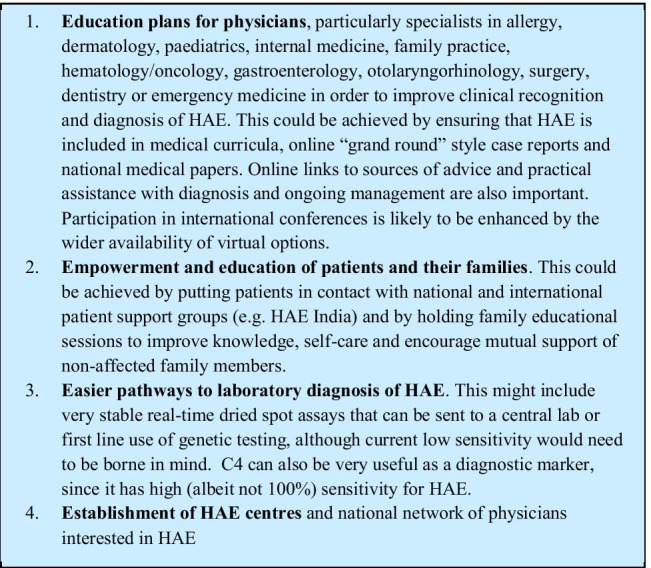



#### Treatment of Acute Attacks

Guidelines state that all attacks should be treated with C1-INH, icatibant or ecallantide [4, 5] and should be treated as early as possible, in order to minimize their duration and severity. Upper airway edema should be treated as a medical emergency. All patients should carry an acute attack treatment on their person and be trained on self-administration (with the exception of ecallantide).

The World Allergy Organization (WAO) guideline and emergency department (ED) guidelines [14] emphasizes the need to consider early intubation or tracheotomy in the case of upper airway angioedema [4]. Intubation or tracheotomy is rarely needed where first-line treatments are available but is likely to be of great importance where this is not the case. Guidelines also suggest that plasma may be used if first-line treatments are not available, depending on local availability and safety [4].

#### India Experience

Management of HAE in India is a daunting task for patients, families and treating physicians. Most patients have to self-support the cost of therapy for management of most diseases, including HAE. There is no universal health insurance, and only a few patients get financial support from their state or central government.

First-line treatments (C1-INH replacement, ecallantide and icatibant) are not available. These drugs may be imported from other countries and may be used on a compassionate basis for individual patients. However, the cost is prohibitively expensive for almost all patients. Options for acute treatment are limited to fresh frozen plasma (FFP) and attenuated androgens which are often used despite lack of scientific evidence for their efficacy in acute treatment.

#### Fresh Frozen Plasma for Management of Attacks

FFP contains approximately 1 unit/ml of C1-INH and is a commonly used treatment for acute angioedema attacks in several countries where plasma-derived C1-INH and icatibant are not available. Fresh frozen plasma in a dosage of 10–15 ml/kg has been shown to be effective to abort episodes of acute HAE in some observational studies, but no controlled trials of this treatment exist [15, 16]. Prematta et al. noted success in 22 of 23 patients with attacks at various locations, including laryngeal involvement, in doses of 1–4 units FFP (approximately 250–1000 U C1-INH) [17]. First improvement was noted at 90 min to 12 h, and resolution occurred between 90 min and > 12 h [17]. In another retrospective hospital-based study carried out in Iran and South Africa, FFP treatment was given for 98 of 176 HAE attacks of which 45 attacks involved the upper airway in 43 patients. Attacks were treated with a median (interquartile range: IQR) dose of 400 ml (range 280–560 ml) of FFP, and episodes resolved after a median (IQR) of 4 h (range 2–12 h). Seven patients required a second treatment; 6 were from South Africa, where the initial treatment dose was lower (median 280 ml) compared with Iran (median 560 ml). Of the 45 laryngeal attacks in 43 patients, seven required intubations. Six of these patients were from South Africa, five of whom were intubated before FFP could be given and two because of lack of FFP response. Amongst the 97 potentially life-threatening upper airway attacks, 45 did not receive FFP treatment [18]. Although FFP was found to be effective in aborting most attacks, the response was slow requiring prolonged hospitalization, resulting in increased overall management costs. Furthermore, in Iran and South Africa, 5% patients experienced transfusion reactions including one severe anaphylactic reaction [18]. Another case series from China describes use of FFP (dose 586 ml ± 387 ml) for 16 acute HAE attacks in 13 patients [19]. All but one patient responded with resolution at 3.3 h (range 2–12 h), compared with historical response times of 61.7 ± 27.0 h. Two patients experienced worsening abdominal pain after infusion and one, who did not improve after FFP, experienced a transfusion reaction.

A recent case report from Pakistan described the first HAE treatment with FFP of a man with facial and laryngeal edema necessitating intubation [20]. He was treated with 20 ml/kg with complete resolution after 18 h.

In addition to transfusion reactions, FFP carries other adverse effects of concern, including transmission of pathogens and volume overload. Decades ago, a theoretical risk of aggravation of an acute attack was postulated, as FFP itself contains contact proteins that may lead to excess bradykinin. However, symptom worsening due to FFP has rarely been reported [17]. No data is available on the risk of blood borne pathogens in Iranian, South African or Chinese patients. However, a US retrospective study noted serological evidence of Hepatitis B, C and HIV in 0.97%, 1.93% and 0.19%, respectively in HAE patients treated with FFP [21].

The optimum dose of FFP is unknown. Based on experience with plasma-derived C1-INH, it seems likely that at least 20 ml/kg is required, although, as reported above, lower doses may still prove lifesaving [22, 23].

The requirement to attend a healthcare facility makes it impossible for many patients to be treated promptly to achieve an optimal response. At times, the HAE attack may be severe enough that patients/parents may not even have enough time to reach the hospital for getting an FFP infusion emphasizing the dire need for an acute “on-demand” self-administered therapy in these countries [9].

Tranexamic acid used acutely during an ongoing attack has been reported to potentially prolong the attack [24], but anecdotally some patients have found that very early treatment during a well-defined predictive prodromal period or very shortly after the onset of an attack may be effective at reducing severity [25, 26]. There is, however, very limited trial data to support this observation [27–30]. Milder attacks (such as peripheral edema and less severe abdominal attacks) may respond to high-dose oral or intravenous tranexamic acid (used in a dose of 1000 mg every 3–4 h for 12–18 h). Likewise, some patients are able to abort attacks by up dosing with attenuated androgens (danazol 200–400 mg or stanozolol 5–7.5 mg) taken immediately upon recognition of prodromal symptoms (e.g. erythema marginatum, severe fatigue or ‘pricking’ sensation) known to result in a subsequent angioedema episode. However, it is widely accepted that androgens are unlikely to contribute to resolution of established attacks.

#### Local Solutions

One of the Indian pharmaceutical companies is currently developing local production of plasma-derived C1-INH, and is awaiting clearance from regulatory authorities. This product is likely to be available on the market by mid-2021. In our experience, completion of the patent period for any particular drug and availability of a locally produced brand leads to drastic fall in the market price of even international Food and Drug Administration (FDA)–approved medications.

We have observed this phenomenon with other analogous high-cost plasma products such as intravenous immunoglobulin (IVIg). At the present time, the cost of locally produced IVIg is at least 3–4 times lower than most international brands, and it is now affordable to patients even for lifelong maintenance treatment. Local production of C1-INH therapy is one of the most effective solutions for patients with HAE in countries like India, as there appears to be no immediate hope of availability and affordability for most other first-line therapies. Given the availability of plentiful C1-INH, a by-product of immunoglobulin and other plasma protein fractionations, we recommend that locally produced C1-INH produced in a formulation and quantity sufficient to provide both acute (intravenous) and prophylactic (subcutaneous) C1-INH replacement at a level expected to provide almost complete freedom from attacks [31]. However, one has to be careful with the safety and efficacy of these plasma-derived products as this is not limited to the actual process of production but also on ‘harvesting’ the raw material and all steps in between. Quality control in this entire process is extremely laborious.

Similarly, now that icatibant, a nonapeptide bradykinin B2 receptor inhibitor, is off patent, there is potential for local production. Although seven generic versions are already available [32–34], the cost of these treatments would be still prohibitive for most patients with HAE in India and other low-income countries.

Contribution from patient-support societies cannot be overemphasized. In India, several states have now started supporting IVIg treatment for patients with primary immunodeficiency diseases. This has been possible because of consistent efforts from The Indian Society for Primary Immune Deficiency (ISPID): a physician society and two patient organizations (viz. Primary Immunodeficiency Patients’ Welfare Society [PIDPWS] and Indian Patients Society for Primary Immunodeficiency [IPSPI]). A similar patient and physician society for HAE in India (https://haei.org/hae-member-countries/india/), affiliated with the HAE International (www.haei.org), has recently been established with an aim to increase awareness about this disease amongst physicians and for providing better access to modern treatments.

HAEi is currently working with pharmaceutical companies in a global access program, which has had success in introducing recombinant C1-INH (Ruconest) into many middle-income countries [35]. Oral rapid-acting kallikrein inhibitors are currently in acute trials [36] and may provide more accessible future options.

We propose (Box 2).

#### Box 2 Proposal to enable better access to effective treatments for acute HAE attacks

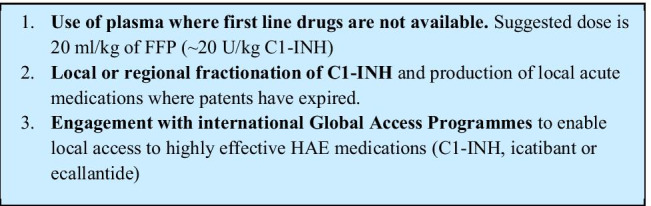



### Long-term Prophylaxis

Guidelines recommend that every patient should be evaluated for long-term prophylaxis [4] and that the highly effective new agents, subcutaneous C1-INH and lanadelumab, should become first-line prophylactic treatments. Guidelines state that attenuated androgens can be effective in long-term prophylaxis (LTP) but that they should be considered second-line, owing to the increased frequency of adverse effects. Guidelines no longer recommend tranexamic acid for LTP, owing to lack of scientific evidence supporting its efficacy. Guidelines strongly emphasize that all patients on LTP should still have access to acute ‘on-demand’ treatment, for breakthrough angioedema episodes ("attacks").

In India and other developing countries, where timely acute treatment is not available, risk-benefit is weighed towards greater use of prophylaxis, despite lack of first-line treatments.

#### Attenuated Androgens for Prophylaxis

Attenuated androgens (danazol, stanozolol, oxandrolone, tibolone) and non-attenuated 17 alpha-alkylated androgens (methyl-testosterone, oxymetholone) are believed to act by increasing intrinsic production of C1-INH.

Double-blind, randomized controlled trials (methyl-testosterone, danazol) and case series (stanozolol, tibolone, oxymetholone, oxandrolone) provide evidence of attenuated androgen effectiveness for LTP in patients with HAE [37]. At high doses, androgens are significantly more effective than placebo in reducing frequency and severity of attacks in more than 90% of patients, including laryngeal attacks [38–46].

However, the side-effect profile of these drugs, especially at doses higher than the equivalent of danazol 200 mg a day, is a limiting factor for use as long-term prophylaxis. Commonly reported side effects include weight gain, acne, virilization, menstrual irregularities, hirsutism, hepatic abnormalities, growth retardation, behavioural and mood alterations, headache and cardiovascular risk [47]. In our preliminary experience from India, these side effects were common (at variable doses of stanozolol and danazol used) [9]. However, no growth abnormalities in children were observed [9, 48].

In view of these findings, attenuated androgen doses should be adjusted to the lowest possible amount consistent with freedom from attacks. The required dose of stanozolol may range from 0.5 mg taken alternate days to up to 4 mg daily or at doses as high as 8 mg a day for short periods throughout the year. Similarly, the dose of danazol may range from 100 mg alternate days to up to 600 mg per day depending on requirements of individual patients. Some patients may benefit from an ‘alternative prophylaxis’ approach with dose adjustments according to known trigger exposure such as menstruation or infections, or even use of androgens intermittently during high-risk periods only [25, 49].

#### Anti-fibrinolytic Agents for Prophylaxis of HAE

Anti-fibrinolytic agents (tranexamic acid in a dose of 30–50 mg/kg/day in 2–3 divided doses, maximum dose 3 g/day) are used for LTP, despite paucity of efficacy data [29, 50–53]. However, better tolerability of anti-fibrinolytic agents compared with attenuated androgens makes them a potential treatment option for LTP especially in children, adolescents and perhaps during pregnancy [37, 51, 54].

There are no studies that have compared the use of attenuated androgens, anti-fibrinolytic agents or a combination of the 2 agents for LTP of HAE. We have used combination of attenuated androgens and tranexamic acid in several of our patients and found no increased risk of thrombosis [9].

#### Other Treatment Options for Prophylaxis

Progestins are not specifically mentioned in the guidelines. However, case series suggest that some, although not all, may have modest prophylactic benefits. Specifically, desogestrel (75–150 µg daily), norethisterone (350 µg–10 mg daily) or medroxyprogesterone acetate (150 mg every 3 months; Depo-Provera) may reduce attack frequency. Higher progestin doses and depot preparations are more likely to be beneficial [55]. Oral progestins are safe to use for HAE female patients requiring ongoing or post-coital contraception, as are intrauterine progestin-eluting contraceptive devices. US HAEA Medical Advisory Board 2020 Guidelines have recommended the use of progestins for HAE-nl-C1-INH [56].

The guideline recommendation that all patients should have access to effective treatment for HAE attacks is problematic in resource-poor countries. Despite beneficial experience of attenuated androgens and tranexamic acid as prophylaxis in India, we recently experienced the death of a 16-year-old girl, who, despite receiving dual prophylaxis with stanozolol and tranexamic acid, had a fatal laryngeal edema. She succumbed before her parents could access a hospital FFP transfusion [9]. In addition, at least five patients recall recent deaths of family members because of similar episodes [9]. Education of patients and families to seek help for facial attacks, which may progress to involve the larynx or for laryngeal attacks immediately after onset, when symptoms are typically mild, may minimize mortality, but is no substitute for effective and accessible at home self-administered treatment.

With the advent of orally available small molecule kallikrein inhibitors, there is a desire on behalf of manufacturers to provide the widest possible access. However, the commercial model for such provision is yet to be determined.

We propose Box 3.

#### Box 3 Proposal to enable better access to prophylactic treatments for hereditary angioedema 

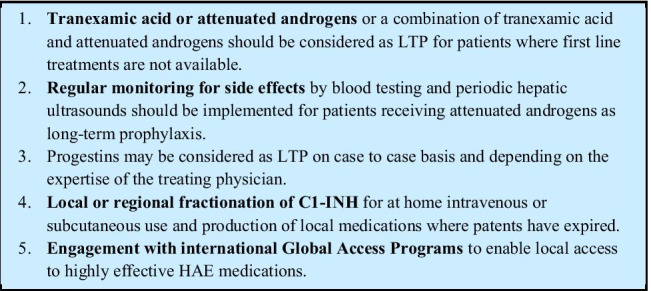



### Short-term Prophylaxis

Guidelines recommend that short-term prophylaxis (STP) should be considered prior to surgical or dental procedures or known patient-specific triggers, and that intravenous pd-C1-INH should be used for STP. The WAO guideline mentions attenuated androgens as second line. The risk-benefit considerations of STP, compared with a strategy of waiting and treating any emergent attack, will be very different in lower-income countries. On the one hand, the risks associated with attacks are greater, while on the other, costs of treatment are more individually and institutionally burdensome.

Retrospective data suggests that the risk of angioedema related to non-dental procedures is relatively low, with one chart-review study of 335 patients finding a 5.7% risk of swelling post-non-dental surgical procedure. Conversely, the risk of angioedema after dental extraction is relatively high, with 21.5% of 577 patients experiencing angioedema [57, 58].

While good evidence exists for efficacy of C1-INH in STP, evidence for plasma, attenuated androgens or anti-fibrinolytics is limited to case series and the optimum dose is unknown. A systematic review of the use of 2–4 units of FFP as STP reported angioedema in only 7 of 148 surgical and dental procedures [17]. With regards to oral agents, androgens appear to be more effective than anti-fibrinolytics [59, 60]. Doses of danazol up to 2.5 to 10 mg/kg or an increase in dose by 50–100% (maximum, 600 mg/day) to be taken daily from 5 days before until 2 to 3 days after the procedure have been recommended in previous guidelines [52]. Tranexamic acid doses of 30–50 mg/kg or a maximum of 3–4.5 g daily in 2–3 divided doses, from up to 5 days before up until 2 days after the procedure has also been suggested [57,61). Sheffer et al. previously reported effective use of tranexamic acid (1 g 6 hourly, from 2 days before, to 2 days after the procedure) as STP for 14/14 patients with HAE (10 undergoing dental extractions and 4 undergoing general surgical procedures) [61]. Eight out of 14 of these patients had previously experienced an angioedema attack of which seven involved the upper airway during dental procedures when no tranexamic prophylaxis was used [61].

Adverse effects appear to be less common than with long-term prophylaxis, and are transient in nature. For this reason, prior to availability of C1-INH, androgens were often used as STP even for children and in the third trimester of pregnancy. In situations of high risk and severe consequences of swelling, we have had STP success with a combination of attenuated androgens and FFP [62].

We propose Box 4.

#### Box 4 Proposals for short term prophylaxis of HAE attacks, where first line therapy is not available

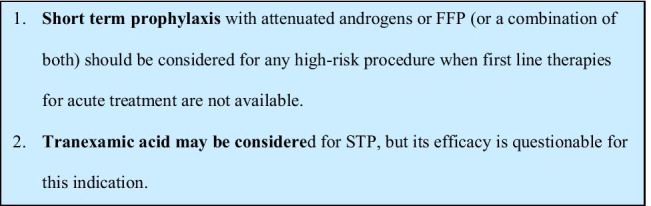



### Special Considerations: Pregnancy, Breastfeeding and Childhood

Global guidelines recommend C1-INH as the only acute and prophylactic treatment in these situations [4, 5, 63]. Icatibant has recently been approved in several countries for acute treatment of HAE attacks in children and is recommended by recent national guidelines [64].

#### Children

In the absence of first-line therapies, FFP is used for acute treatment in a dose of 10 ml/kg and tranexamic acid for STP (at a maximum dose of 15–25 mg/kg twice or three times daily), adjusted for gastrointestinal tolerability and efficacy [63].

Since a greater proportion of children are attack-free compared with adults, the need for LTP is less frequent. Nevertheless, some children are severely affected and if tranexamic acid is insufficient, and first-line therapies are unavailable, attenuated androgens may be unavoidable. While guidelines advise against androgen use prior to Tanner Stage V of puberty, older case reports and series do advocate their use [65, 66]. In view of the potential for adverse effects, the dose should be adjusted to the minimum amount consistent with reasonable freedom from severe attacks and after correction of any underlying precipitating factors. A Hungarian centre describes good tolerability of danazol used as LTP over a period of 2–6 years with a dose of 100 mg every 2–6 days, albeit they did not specify the age group that was treated [26, 65, 66].

Indian experience suggests that while patients experience reductions in attack frequency, adverse effects are common, with virilization and precocious puberty being of particular concern [9, 67]. However, growth retardation appears to be relatively infrequent [9, 48].

We propose Box 5, for children.

#### Box 5 Proposals for HAE treatment for Children, where first line treatments are not available.^1^

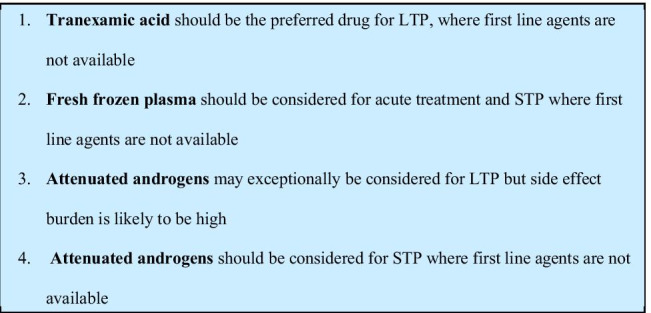



#### Pregnancy and Breastfeeding

Attenuated androgens are potentially teratogenic, with potential to masculinize the female foetus. They may reduce but not completely abolish the ability to conceive and are contraindicated during pregnancy and during breastfeeding. Case reports cite androgen use during pregnancy, without maternal or foetal injury but evidence of safety is lacking [68–70]. We have reported a case of successful breastfeeding despite danazol use [62], although attenuated androgens may be present in small quantities in breast milk.

Case reports suggest that tranexamic acid may be helpful for HAE during pregnancy but evidence of efficacy and safety, particularly with respect to lack of teratogenicity or thrombosis risk, is lacking. Where C1-INH is not available, tranexamic acid could be considered, preferably after the first trimester [68, 71–73]. A single case report cites the use of FFP as LTP during pregnancy [74].

We propose that in pregnancy (Box 6).

#### Box 6 Proposals for HAE management in pregnancy, where first line treatments are not available

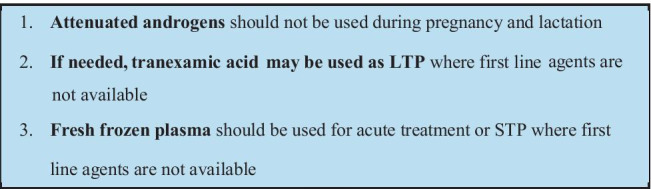



#### Avoidance of Triggers

The WAO guideline recommends that all patients with HAE should be educated about possible triggers, which may induce HAE attacks [4].

Although the trigger is not evident for the majority of HAE attacks, this recommendation is particularly important where recommended acute attack treatments are not available. Angiotensin convertase (ACE) inhibitors and estrogens are known to be major triggers and should be avoided by all patients [28, 75, 76]. Chronic infections such as *Helicobacter pylori* [77] or inflammatory conditions such as poor dental health [4] or celiac disease [78] or associated immunoregulatory disorders [79] may lower the threshold for swelling. We suggest that these obvious as well as more subtle triggers be proactively identified and treated whenever possible and to encourage preventive medicine including routine dental work.

In addition, it is also important for low-income countries to have an HAE patient organization and to find physicians who are interested and are willing to inform the appropriate healthcare authorities for improving the situation of HAE in the country. This may include reference to other countries with similar income but who have better treatment options for HAE. Contacting HAEi and pharmaceutical companies and using the experience of other countries in a similar situation could be extremely useful.

## Conclusions

Results of our review suggest that there are significant inequities in provision of HAE services and treatments throughout the world. HAE patients in low-income countries do not have access to life-saving acute medications or recently developed highly effective LTP medications. Many countries have very limited access to older LTP treatments. Most low-income countries do not have access to HAE specialists and related services or diagnostic facilities, resulting in long delays or missed diagnosis.

We propose suggestions to optimize the use of limited resources which could be used as the starting point for future discussion and consensus. However, this document underscores the urgent need to improve HAE services, diagnostics and treatment access to lower-income countries. We call upon all HAE stakeholders to support the cause of global equity and access to these life-saving measures.

## Supplementary Information

Below is the link to the electronic supplementary material.

Supplementary file1 (DOCX 77 KB)

## Abbreviations

*ACE-I*: Angiotensin-converting enzyme inhibitors
*GDP*: Gross domestic product
*C1-INH*: C1 inhibitor
*C1-INH-HAE*: Hereditary angioedema with C1 inhibitor deficiency
*FFP*: Fresh-frozen plasma
*HAE*: Hereditary angioedema
*IVIg*: Intravenous immunoglobulins
*LTP*: Long-term prophylaxis
*PGIMER*: Postgraduate Institute of Medical Education and Research (India)
*STP*: Short-term prophylaxis
*TA*: Tranexamic acid

## Footnotes

1 Attenuated androgens may exceptionally be considered for LTP but side effect burden is likely to be high
