# Correction to: Mitigating Disparity in Healthcare Resources Between Countries for Management of Hereditary Angioedema

**DOI:** 10.1007/s12016-021-08869-y

**Published:** 2021-07-01

**Authors:** Ankur Kumar Jindal, Avner Reshef, Hilary Longhurst, GEHM workgroup (Global Equity in H. A. E. Management)

**Affiliations:** 1 Allergy Immunology Unit, Department of Paediatrics, Advanced Paediatrics Centre, Postgraduate Institute of Medical Education and Research, Chandigarh, India; 2 Allergy Immunology & Angioedema Center, Barzilai University Medical Center, Ashkelon, Israel; 3 Auckland District Health Board, Auckland, New Zealand; 4 Department of Allergy and Immunology, University College Hospitals, London, England; 5 Department of Medicine, University of Auckland, Auckland, New Zealand

## Correction to: Clinical Reviews in Allergy & Immunology: 10.1007/s12016-021-08854-5

The original version of this article unfortunately contained a mistake. We need to add a contributor to Table 1, who contributed to the paper, in particular the data collection and whose name was left off the final list of contributors. The corrected Table 1 is given below. The original article has been corrected.

**Table 1:** List of the Global Equity in HAE Management (GEHM) workgroup participants

	Country	Name		Affiliation	Mail
1	Austria	Aberer	Werner	Medical University of Graz, Graz, Austria	werner.aberer@medunigraz.at
2	Canada	Betchel	Stephen	Univ. of Toronto, St. Michael's Hospital, Toronto, Canada	Stephen.Betschel@unityhealth.to
3	Germany	Bork Aygören-Pürsün Maurer Magerl	Konrad Emel Marcus Markus	Universitäts-Hautklinik, Dermatology, Mainz, Germany Goethe-Universität, Frankfurt am Main, Germany Charité - Universitätsmedizin Berlin, Berlin, Germany Charité - Universitätsmedizin Berlin, Berlin, Germany	konrad.bork@unimedizin-mainz.de aygoeren@em.uni-frankfurt.de marcus.maurer@charite.de markus.magerl@charite.de
4	France	Bouillet	Laurence	Chercheur à Université Grenoble Alpes, Grenoble, France	LBouillet@chu-grenoble.fr
5	Denmark	Bygum	Anette	Odense Universitetshospital, Odense, Denmark	Anette.Bygum@rsyd.dk
6	Spain	Caballero	Teresa	Hospital Universitario La Paz, Madrid, Spain	tercaballero@gmail.com
7	Italy	Cancian	Mauro	University Hospital of Padova, Padova, Italy	mcancian@unipd.it
8	Hungary	Farkas	Henriette	Angioedema Ctr, Semmelweis University, Budapest, Hungary	farkas.henriette@med.semmelweis-univ.hu
9	North Macedonia	Grivcheva-Panovska	Vesna	University Sts Cyril and Methodius Skopje, North Macedonia	vesna_grivcheva_panovska@yahoo.com
10	Brazil	Grumach	Anete	Ctr for Rare Diseases, Faculdade de Medicina, São Paulo, Brazil	anete@grumach.com
11	Turkey	Gulbahar	Okan	Ege Üniversitesi, Izmir, Turkey	okan.gulbahar@yahoo.com
12	Japan	Hide	Michihiro	Dept of Dermatology, Hiroshima Univ. Hiroshima, Japan	ed1h-w1de-road@hiroshima-u.ac.jp
13	India	Jindal Singh	Ankur Surjit	Postgraduate Institute of Medical Education and Research, Chandigarh, India Postgraduate Institute of Medical Education and Research, Chandigarh, India	ankurjindal11@gmail.com surjitsinghpgi@rediffmail.com
14	Bangladesh	Jindal	Ankur	Postgraduate Institute of Medical Education and Research, Chandigarh, India	ankurjindal11@gmail.com
15	South Korea	Kang	Hye-Ryun	Seoul National University Hospital, Seoul, South Korea	helenmed@snu.ac.kr
16	Israel	Reshef Kessel	Avner Aharon	Barzilai University Medical Ctr, Ashkelon, Israel Bnay-Zion Med Ctr, Technion Medical School, Haifa, Israel	avnerre@bmc.gov.il aharon.kessel@b-zion.org.il
17	United Kingdom	Longhurst	Hilary	UCLH, London, UK Department of Medicine, University of Auckland, New Zealand	hlonghurst@doctors.org.uk
18	New Zealand	Lindsay Jordan Ameratunga	Karen Anthony Rohan	Auckland District Health Board Auckland District Health Board Department of Molecular Medicine and Pathology Faculty of Medical and Health Sciences, University of Auckland	KLindsay@adhb.govt.nz AnthonyJ@adhb.govt.nz rame001@aucklanduni.ac.nz
19	USA	Lumry Bernstein Craig Riedl Levy	William Jonathan Timothy Marc Don	Allergy & Immunology Assoc., Dallas TX, USA Univ. Cincinnati, Div. of Immunology, Cincinnati OH, USA Penn State University Hershey, PA, USA US HAEA Angioedema Ctr, Univ. of California, San Diego CA USA University of California at Irvine, Irvine, CA, USA	Lumrymd@me.com BERNSTJA@ucmail.uc.edu tcraig@pennstatehealth.psu.edu mriedl@health.ucsd.edu DLevy1@uci.edu
20	Argentina	Malbran	Alejandro	Asociación Argentina de AH, Buenos Aires, Argentina	amalbran31@hotmail.com
21	Greece	Germenis Psarros	Anastasios Fotis	School of Medicine, University of Thessaly, Larissa, Greece Greek Navy Hospital, Athens, Greece	agermen@med.uth.gr psarros@allergy.gr
22	Poland	Stobiecki Porebski	Marcin Grzegorz	Jagelonian University, Krakow, Poland Department of Clinical and Environmental Allergology, Jagiellonian University Medical College ul. Botaniczna 3, Krakow, Poland	marcin.stobiecki@uj.edu.pl g.porebski@uj.edu.pl
23	Bulgaria	Valerieva	Anna	University Hospital “Alexandrovska”, Sofia, Bulgaria	anna.valerieva@gmail.com
24 25 26	Australia Indonesia Hong Kong	Wardman	Fiona	HAE International (HAEi), Chief Regional Patient Advocate and Regional Patient Advocate, Asia Pacific	f.wardman@haei.org
27	Singapore	Wardman Zhong	Fiona Youjia	HAE International (HAEi), Chief Regional Patient Advocate and Regional Patient Advocate, Asia Pacific National University Hospital, Singapore	f.wardman@haei.org youjia_zhong@nuhs.edu.sg
28	Switzerland	Weber	Christina	Allergiestation, Universitätsspital Zürich, Switzerland	weber@chinderarztpraxis.ch

