# Supplementary material for: Mitigating Disparity in Health-care Resources Between Countries for Management of Hereditary Angioedema
Source: Clin Rev Allergy Immunol. 2021 May 18;61(1):84–97. doi: 10.1007/s12016-021-08854-5 (PMC8282575; doi:10.1007/s12016-021-08854-5)
Supplement: Supplementary file 1 — Supplementary file1 (DOCX 77 KB) [file 12016_2021_8854_MOESM1_ESM.docx]

**Supplementary Table 3. Country-by-country demography, income, access to therapy and specific unmet needs for management of HAE**

| **Country** | **Poland** | **India^** | **United Kingdom** | **New Zealand** | **Italy^^** | **Bulgaria** | **France** | **Turkey^^^** |
| --- | --- | --- | --- | --- | --- | --- | --- | --- |
| **Continent** | Europe | Asia | Europe | Australasia | Europe | Europe | Europe | Europe |
| **Population (rounded)** | 37,850,000 | 1,380,000,000 | 60,000,000 | 5,000,000 | 60,250,000 | 7,000,000 | 67,000,000 | 83,150.000 |
| **GDP (per capita USD)^1^** | 15,420.91 | 2,009.98 | 42,943.90 | 41,945.33 | 34,483.20 | 9,272.63 | 41,463.64 | 9,370.18 |
| **Estimated diagnosed HAE** | 400 adults  30 children | 100 adults  50 children | 600 | 50 adults  3 children | 900 adults  80 children | 80 adults  12 children | 1,500 | 600 adults  100 children |
| **Management guidelines** | Yes^2^ | No | Yes**^3^** | Yes**^4^** | Yes^5^ | WAO/EAACI^6^  Local algorithms | French National Diagnosis and Treatment Protocol (PNDS) | WAO/EAACI Guidelines^6^ |
| **Customary acute (OD) treatments** | Icatibant, rhC1-INH (Ruconest; temporarily unavailable), pd C1-INH (Berinert) | FFP | pdC1-INH (Berinert, Cinryze), rhC1-INH (Ruconest), icatibant | pdC1-INH (Berinert, Cinryze), rhC1-INH (Ruconest), icatibant | Icatibant, iv pd C1-INH (Berinert, Cinryze), iv rhC1-INH (Ruconest)# | pd C1-INH (Berinert), rhC1-INH (Ruconest) and icatibant | pdC1-INH (Berinert, Cinryze), rhC1-INH (Ruconest), icatibant | Icatibant -Self, SC pdC1-INH (Cinryze)-at hospitals, IV  FFP |
| **Customary prophylactic (STP, LTP) treatments** | STP: Berinert  LTP: Danazol, TA, FFP | STP: Stanozolol, danazol, FFP  LTP: Stanozolol, danazol, TA | STP: Berinert, Cinryze, Ruconest  LTP: Danazol (limited availability), oxandrolone, TA, pdC1-INH iv only*, lanadelumab*  *only if >2 attacks per week | STP: Berinert, Cinryze, Ruconest  LTP: Danazol, Stanozolol, TA, pdC1-INH iv only*,  *> 8 attacks per month | STP: oral danazol, iv pdC1-INH (Berinert, Cinryze)  LTP: oral danazol, iv pdC1-INH Cinryze, ## sc Lanadelumab## | STP: Berinert, Ruconest  LTP - no options available | STP: Berinert, Cinryze, Ruconest  LTP: Ruconest, Cinryze, Berinert, lanadelumab, danazol, TA | STP: Cinryze  Danazol  FFP  LTP: Cinryze  Danazol  Tranexamic acid  (TA) |
| **What drugs are available?**  **What is registered?** | Icatibant (Firazyr), Ruconest (temporary unavailable), Berinert  Available but not registered: Danazol | Available but not registered: Stanozolol, Danazol, TA, FFP | Available: as above  Registered: as above, except oxandrolone. | pdC1-INH sc and lanadelumab registered but not funded and not available. | Available and registered as above | C1-INHs (Berinert, Ruconest registered and available)  - Icatibant registered and available  - FFP is available but generally not used, only in medical facilities where C1-INH and icatibant are not available  - TA is not available, patients can have it from Greece (lately only a tablet of 250 mg  - Androgens - not available | All are available  Registered:  Acute:  Icatibant  Berinert  Cinryze  Ruconest  STP:  Berinert  Cinryze  LTP:  Danazol  Cinryze  Lanadelumab | All are available and registered as above |
| **Access to medications** | Firazyr, Ruconest (temporary unavailable), Berinert – all reimbursed by National Health Fund | Largely self-funded | Funded by National Health Service | Funded nationally (Pharmac) | Funded by NHS### | Access to HAE medication is regulated through a protocol prescription process of the National Health Fund (NHF). Protocols are renewed every 6 months after a follow-up visit, follow-up blood tests, and when the medication prescribed for the last period have been used by the patient (patient diary is applied together with medical documents); Costs for drugs are covered 100% by the NHF. | All reimbursed by national health assurance | Funded by National Health Insurance:  Icatibant (Bradikant, Icatin)  Cinryze (Acute treatment, and short term-prophylaxis for surgery. Special permission required for LTP, or may be self-funded)  Danazol  TA  FFP |
| **Cost to patient (if available)** | Berinert- patient co-payment ca. 1EUR/ package (available on prescription at the pharmacy) | Danazol: 4-10 USD for 10 tablets, 100 mg each  Stanozolol: 2-4 USD for 10 tables, 2 mg each  TA: 2-5 USD for 10 tablets, 500 mg each | £7 per prescription for oral treatments (usually 3 month’s supply) | NZ$5 per item prescription (usually 3 months) | Approximately 1200-2400 $ for OD treatment, depending on the drug chosen, the patient's weight and posology  STP: 10 $ for danazol, around 1200-1750 $ for pdC1-INH depending on the drug chosen  LTP: 25 $ a month for danazol, around 15-18.000 $ a month for sc or iv prophylaxis, depending on the drug chosen and interval of administration. | - pdC1-INH (Berinert 500 U, 10 ml) for intravenous application: 1263 Bulgarian leva (BGN) / vial (approximately ~ 645 EUR/vial)  - rhC1-INH (Ruconest 2100 U, 14 ml) for intravenous application: 1660 BGN / vial (~ 848 EUR/vial)  - icatibant (3 ml) for subcutaneous application: 3935 BGN / vial (~ 2010 EUR/vial) (a form with 3 vials is the only available with a price of 11806 BGN ) | No charge for patient | No charge for patient  Cinryze: 607 $  Danazol 100 mg/100 capsules: 16$  TA 500 mg/50 tablets: 6$  Icatibant:  Bradikant: 385 $  Icatin: 390 $ |
| **Insurance, health plans, government support/co-payment, compassionate-use programs** | Most patients are insured in National Health. It covers costs of medications, diagnostic procedures and emergency tx including hospitalization | Few patients get their treatment supported from Government | Universal government coverage.  Compassionate use for Berotralstat planned. | Universal government coverage | Universal government coverage | National Health Fund pays 100% of drug costs for patients with active health insurance | Universal government coverage | Universal government coverage |
| **Generic versions** | Icatibant | No | No | No | No | No | No | Icatibant (Bradikant, Icatin) |
| **Local production** | FFP | FFP, C1-INH (in process) | FFP | FFP | FFP | No | none | FFP,Danazol  TA, Bradikant, Icatin (Active ingredients are imported) |

| **Country** | **Denmark** | **Hungary^^^^** | **Israel** | **North Macedonia^^^^^** | **Canada** | **Greece** |
| --- | --- | --- | --- | --- | --- | --- |
| **Continent** | **Europe** | **Europe** | **Asia** | **Europe** | **North America** | **Europe** |
| **Population (rounded)** | 5,800,000 | 9,650,000 | 9,136,000 | 2,000,000 | 37,100,000 | 10,700,000 |
| **GDP (per capita USD)^1^** | 61,350.35 | 16,161.98 | 41,715.03 | 6,083.72 | 46,232.99 | 20,324.25 |
| **Estimated diagnosed HAE patients** | 90 adults  22 children | 170 adults  28 children | 250-300 (about 1/3 children & adolescents) | 36 adults 4 children | 800 | 179 (approximately 10% children) |
| **Management guidelines** | Yes | Local^7^ and current international^8^ guidelines | Yes^9^ | WAO/EAACI guidelines^6^ | Yes (Canadian/International)^8^ | WAO/EAACI guidelines^6^ |
| **Customary acute (OD) treatments** | pd C1-INH (Berinert, Cinryze), rhC1-INH (Ruconest), Icatibant | pdC1-INH (Berinert), rhC1-INH (Ruconest), icatibant | pdC1-INH (Berinert), rhC1-INH (Ruconest), icatibant Icatibant Teva (generic) | pdC1-INH (Berinert), rhC1-INH (Ruconest) | pdC1-INH (Berinert), icatibant rarely FFP | pdC1-INH (Berinert), icatibant |
| **Customary prophylactic (STP, LTP) treatments** | STP: Berinert, Cinryze, Ruconest  LTP: Danazol, TA, pdC1-INH iv or sc, lanadelumab*  *At least 4 attacks per month or severely reduced QoL (after special permission) | STP:  Berinert iv., Danazol  LTP:  Danazol,  TA  Berinert iv and sc,  Lanadelumab | STP: Berinert, Ruconest  LTP: Danazol, TA, Lanadelumab | STP: Berinert, Ruconest  LTP: Not available | LTP:  pdC1-INH IV and SC  Danazol rarely  Lanadelumab on compassionate for now (licensed and awaiting reimbursement decisions with private payers and public payers) | STP:  1st choice Berinert, 2nd choice Danazol  LTP:  Danazol, TA, Progestins, Berinert i.v. (2 patients), C1-INH sc (1 patient), Lanadelumab (9 patients) |
| **What drugs are available?**  **What is registered?** | Available as above  Danazol not registered/licensed  TA not registered/licensed for HAE | Available: as above, except Danazol (name-based import permit)  Registered: as above, except TA and Berinert iv. for LTP | Berinert, Ruconest, Lanadelumab, Icatibant, Lanadelumab- co-payed and available  Danazol, TA -covered by the Basic health basket. | Available and registered: Berinert  Available but not registered: Ruconest | Available: danazol, TA, FFP, pd C1-INH IV and SC, Lanadelumab, icatibant  Has indication: all except danazol which is used off label | Berinert, Firazyr, Danazol, TA, FFP and Lanadelumab |
| **Access to medications** | Funded by regions  However, co-payment for TA and danazol | Reimbursed by the National Health Insurance Fund Administration (NEAK), accessible on prescription via pharmacies. | Co-payed by the National Extended Health Basket | Funded by Ministry of Health (Program for Rare Diseases) | Covered for all: pd C1-INH IV and SC, FFP  Covered through private and public: icatibant (private and provincial plans with different criteria for each province; Lanadelumab being negotiated; danazol and TA private and public provincial plans | The newest drugs are very expensive but do not burden the patient, as they are covered by the Greek state |
| **Cost to patient(if available)** | 489 dkr per 100 tablets of TA | Berinert iv, Ruconest, Firazyr 100% reimbursed, patient co-payment ca. 1 EUR/package. Oral LTPs: Danazol 90%, TA 50% reimbursed. Parenteral LTPs: under negotiation. | Qualified HAE patients (diagnosed Type I-II) pay quarterly ~290$ for any amount of prescriptions | Cost free for patients (fully funded by Ministry of health) | Cost free for patients | Cost free for patients |
| **Insurance, health plans, government support/co-payment, compassionate-use programs** | Government coverage for specific HAE medications | All patients are insured in National Health. It covers costs of medications, diagnostic procedures and emergency treatment including hospitalization | Universal government coverage (Basic health basket) provided by 4 HMO's  Additional private insurance packages are also available | Universal government coverage for medications, diagnostic procedures and emergency treatment including hospitalization | All blood products in Canada are 'free' with few criteria to prescribe; provinces decide to list drugs independently for public coverage but only if no private coverage and must meet criteria for coverage; private plans cover most drugs but general rule is 1/3 have private, 1/3 qualify for government, 1/3 pay out of pocket but for these expensive medication almost all would qualify for government coverage.  Landadelumab : $ cdn10,000 / dose (300mg) | Lanadelumab require a specific procedure. National drug reimbursement involves a patient by patient request by an expert based on unmet need for this patient. |
| **Generic versions** | Icatibant | None | Icatibant Teva | N/A | No | No |
| **Local production** | FFP (but not used any longer) | None | FFP | N/A | No | No |

| **Country** | **Austria** | **Japan** | **Germany** | **United States** | **Brazil** | **Argentina** | **Spain^^^^^^** |
| --- | --- | --- | --- | --- | --- | --- | --- |
| **Continent** | **Europe** | **Asia** | **Europe** | **North America** | **Latin America** | **Latin America** | **Europe** |
| **Population (rounded)** | 8,900,000 | 125,800,000 | 83,000,000 | 328,000,000 | 209,000,000 | 45,000,000 | 46,800,000 |
| **GDP (per capita USD)^1^** | 51,461.95 | 39,289.96 | 47,603.03 | 62,794.59 | 8,920.76 | 11,683.95 | 30,370.89 |
| **Estimated diagnosed HAE patients** | 120 | 450 patients | 1800 adults  and children | 6500 | 1,000 with HAE 1/2 | >500 | 900 adults  100 children |
| **Management guidelines** | WAO/EAACI guidelines^6^ | Yes^10,11^ | Yes^12^ | WAO/EAACI | WAO/EAACI | Yes^13, 14^ | Yes^15,16^ |
| **Customary acute (OD) treatments** | pdC1-INH (Berinert, Cinryze), rhC1-INH (Ruconest), icatibant | pdC1-INH (Berinert), icatibant | pdC1-INH (Berinert, Cinryze), rhC1-INH (Ruconest), icatibant | pdC1-INH (Berinert), rhC1-INH (Ruconest), icatibant, Ecallantide | Icatibant, pd C1-INH (Berinert) (not supported or reimbursed by public or private insurance) | Icatibant and C1-INH concentrate | pdC1-INH (Berinert, Cinryze), icatibant |
| **Customary prophylactic (STP, LTP) treatments** | STP: Berinert, Cinryze, Ruconest, Danazol  LTP: Danazol; pdC1-INH i.v. (Berinert, Cinryze) and s.c. (Berinert), Ruconest, lanadelumab | STP: Berinert,  LTP: Danazol, TA | STP: Berinert, Cinryze  LTP: pdC1-INH iv (Cinryze), lanadelumab, pdC1-INH sc (Berinert), danazol, oxandrolone, TA | STP: Berinert, Danazol  LTP: Danazol, pdC1NH (Cinryze, Haegarda), lanadelumab, TA | STP: Berinert (not supported or reimbursed by public insurance)  LTP: Danazol, Oxandrolone, TA, FFP | STP and LTP: danazol, TA, icatibant and pd C1-INH concentrate | STP: danazol, pd C1-INH (Berinert, Cinryze)  LTP: danazol, estanozolol, TA, pd C1-INH (IV), pd C1-INH (SC) |
| **What drugs are available?**  **What is registered?** | Available: as above  Registered: as above | Available: as above  Registered: Berinert, icatibant | Available: as above  Registered: as above, except Danazol, oxandrolone | Registered and Available: as above | Icatibant, pdC1-INH (Berinert)  Lanadelumab: Registered but not supported or reimbursed by public or private insurance. | All drugs are available and registered | Available: danazol, estanozolol, TA, pd C1-INH iv (Berinert, Cinryze), pd C1-INH sc (Berinert), icatibant  Available, not commercialized: estanozolol |
| **Access to medications** | Funded by Health Insurance (for everybody in need) | Largely funded by National Health Insurance. | Coverage by social and private insurances | Access available for all with some private insurance limitation | Danazol funded by National Health Service (only one) | Drugs are accessible to 90% patients, though access is difficult and sometimes through a lot of creativity. One member of a kindred has access and shares with other members on a First-In-First-Out (FIFO) basis | Danazol, tranexamic acid (partially funded by National Health System)  Estanozolol (not funded)  Berinert (iv, sc), Cinryze (iv), icatibant (sc) (fully funded by National Health System, but through hospital budget) |
| **Cost to patient (if available)** | 0.5 €/ prescription | Maximum 2,500 JPY to 30,000 JPY per month for patients, depending on the income. |  | Funded by private and government insurance and foundations | Berinert (patient payment US $400/500 IU/mL)  Icatibant (US $1,800/syringe)  Lanadelumab (US$13,000/syringe) |  | Berinert 500U (iv) 609.35 €  Berinert 1500U (iv) 1711.75 €  Patient pays fully for stanozolol Eu52.59 for 70 x 2mg.  Most patients are insured by National Health System. It covers costs of new HAE specific medications, diagnostic procedures and emergency treatment including hospitalization  Private insurances usually do not cover drug cost. |
| **Insurance, health plans, government support/co-payment, compassionate-use programs** | All patients are insured in National Health. It covers costs of medications, diagnostic procedures and emergency treatment including hospitalization | Universal government support/ co-payment. Upper limit settings for the co-payment. The setting may vary, depending on the frequency of high cost payments, and annual income of the patient. | Coverage by social and private insurances | Berinert, Ruconest, ecallantide $8000-$14000/dose Icatibant $6500/dose  Cinryze $500,000/yr/Lanadelumab $300,000-600,000/yr  Haegarda $400,000-$600,000/yr | Private insurance support STP, sometimes.  No support for other therapies except for danazol | C1-INH concentrate has government support and is free for most patients within social security but not the icatibant. | Berinert 2000U (sc) 2262.95€  Cinryze 1000U (iv) 1160.55€  Firazyr 30 mg (sc) 1697.55€  Danatrol 50 mg (60 capsules) 10.4€  Danatrol 100 mg (60 capsules) 20.14€  Danatrol 200 mg (60 capsules) 39.34€  Amchafibrin® 500 mg (30 pills) 4.62 €  Stanozolol (not commercialized, but available in special pharmacies under physician prescription): 70 pills, 52.59 €  Patient does not copay Berinert, Cinryze or Firazyr.  Patient copays a variable percentage of Danazol and TA according to his/her income (30-50%) |
| **Generic versions** | No | No |  | Icatibant | No | No | No |
| **Local production** | FFP, but not in use any more | NA | FFP | FFP, pdC1-INH, icatibant, ecallantide, lanadelumab | FFP | No | TA |

| **Country** | **South Korea** | **Bangladesh^^^^^^^** | **Australia** | **Indonesia** | **Singapore** | **Hong Kong** | **Switzerland** |
| --- | --- | --- | --- | --- | --- | --- | --- |
| **Continent** | **Asia** | **Asia** | **Australasia** | **South East Asia** | **South East Asia** | **Asia** | **Europe** |
| **Population (rounded)** | 51,780,000 | 161,400,000 | 26,000,000 | 273,525,000 | 5,900,000 | 7,500,000 | 8,500,000 |
| **GDP (per capita USD)^1^** | 31,362.75 | 1,698.26 | 54,200 | 970 | 340 | 275 | 82796.55 |
| **Estimated diagnosed HAE patients** | 70 | 10 | 250 adults  20 children | <5 adults  0 children | <10 adults  <5 children | <20 adults  <5 children | 150 |
| **Management guidelines** | No | No | Yes**^4^** | No | No | No | WAO/EAACI |
| **Customary acute (OD) treatments** | Icatibant, FFP | FFP | Icatibant, pd C1-INH (Berinert) | FFP | FFP, C1-INH | FFP, C1-INH | Pd C1-INH (Berinert, Cinryze), Icatibant |
| **Customary prophylactic (STP, LTP) treatments** | STP: Danazol  LTP: Danazol, TA | STP: Danazol, FFP  LTP: Danazol, TA | pdC1-INH (Berinert iv), pdC1-INH (Berinert sc), TA,  (Danazol no longer available) | None | Danazol, TA | Danazol, TA | STP: pd C1-INH  LTP: pd C1-INH, lanadelumab, danazol |
| **What drugs are available?**  **What is registered?** | Available: as above Registered: Icatibant | Danazol, TA, FFP | As above  Lanadelumab registered and going through the process of funding. | None | pd C1-INH iv, Danazol, TA | pd C1-INH (restricted amount) | Cinryze, Berinert, Icatibant and lanadelumab |
| **Access to medications** | Funded by National Health Insurance | Not funded | pdC1-INH (iv and sc) funded by the National Blood Authority (for patients fulfilling the criteria)  Icatibant funded on PBS (Pharmaceutical Benefits Scheme) | Self-funded | Common medications funded | Self-funded | Cinryze, Berinert and icatibant paid by the health insurance. Lanadelumab (about 12,000 CHF), we have to ask the insurance, not always paid, but registered in Switzerland |
| **Cost to patient (if available)** | Danazol 25,000-30,000 KRW per month Firazyr 1,035,000 KRW/syringe (Patients pay 10% of the cost) | TA: 20-30 USD per month  Danazol: 10-30 USD per month  Patients have to pay commercial cost | Patients pay a dispensing fee only | Patients have to pay commercial cost | Variable |  |  |
| **Insurance, health plans, government support/co-payment, compassionate-use programs** | Universal government support/ co-payment of medical & hospital-related costs according to the reimbursement guidelines. Upper limit of patient payment is set and varies depending on incomes and assets. | Patients have to pay commercial cost | pdC1-INH (iv and sc) funded by the National Blood Authority for patients with 8 attacks or more a month.  Patients pay a dispensing fee from pharmacy for TA and Icatibant of up to $ 41 for most medicines ($ 6.60 if on concession card) |  | Universal health system |  |  |
| **Generic versions** |  | None | Icatibant |  |  |  |  |
| **Local production** | FFP | Nil | FFP |  | FFP | FFP | Nil |

**Footnotes:**

1. <https://datacommons.org/place/country/>
2. <https://www.termedia.pl/Management-of-hereditary-angioedema-with-C1-inhibitor-deficiency-consensus-statement-of-the-HAE-Section-of-the-Polish-Society-of-Allergology-Part-I-classification-pathophysiology-clinical-symptoms-and,123,33173,0,1.html> and <https://www.termedia.pl/Management-of-hereditary-angioedema-with-C1-inhibitor-deficiency-consensus-statement-of-the-HAE-Section-of-the-Polish-Society-of-Allergology-Part-II-treatment-follow-up-and-special-situations,123,33174,0,1.html>
3. Longhurst HJ, Tarzi MD, Ashworth F, Bethune C, Cale C, Dempster J, Gompels M, Jolles S, Seneviratne S, Symons C, Price A, Edgar D. C1 inhibitor deficiency: 2014 United Kingdom consensus document. Clin Exp Immunol. 2015 Jun;180(3):475-83. doi: 10.1111/cei.12584.
4. <https://www.allergy.org.au/images/stories/pospapers/ASCIA_HP_Position_Paper_HAE_2020_Aug_Update.pdf>
5. Mauro Cancian, Italian network for C1-INH-HAE (ITACA). Diagnostic and therapeutic management of hereditary angioedema due to C1-inhibitor deficiency: the Italian experience. Curr Opin Allergy Clin Immunol 2015; 15(4):383-91. doi: 10.1097/ACI.0000000000000186.
6. Maurer M, Magerl M, Ansotegui I, Aygören-Pürsün E, Betschel S, Bork K, Bowen T, Balle Boysen H, Farkas H, Grumach AS, Hide M, Katelaris C, Lockey R, Longhurst H, Lumry WR, Martinez-Saguer I, Moldovan D, Nast A, Pawankar R, Potter P, Riedl M, Ritchie B, Rosenwasser L, Sánchez-Borges M, Zhi Y, Zuraw B, Craig T. The international WAO/EAACI guideline for the management of hereditary angioedema-The 2017 revision and update. Allergy. 2018 Aug;73(8):1575-1596. doi: 10.1111/all.13384.
7. Hungarian College of Clinical Immunology and Allergology. [Ministry of National Resources (NEFMI) professional guideline on the allergologic emergencies – diagnosis and treatment of anaphylaxis, insect venom allergy and hereditary angioedema (Version 1, amended).] (in Hungarian) *Eü. Közlöny [Health Gazette].* 2010 Sep 27;60(18):2821-2835
8. Betschel S, Badiou J, Binkley K, et al. The International/Canadian Hereditary Angioedema Guideline. *Allergy Asthma Clin Immunol.* 2019 Nov 25;15:72. doi: 10.1186/s13223-019-0376-8. eCollection 2019
9. <https://www.ima.org.il/mainsitenew/ViewCategory.aspx?CategoryId=3198> 2009 (Hebrew, Not updated)
10. http://square.umin.ac.jp/compl/common/images/disease-information/hae/HAEGuideline2019_3...pdf
11. WAO Guideline for the management of hereditary angioedema was translated into Japanese and published.
12. https://doi.org/10.1007/s40629-018-0088-5
13. Malbrán A, Malbrán E, Menéndez A, Fernández Romero DS. Angioedema hereditario. Tratamiento del ataque agudo en la Argentina [Hereditary angioedema. Treatment of acute attacks in Argentina]. Medicina (B Aires). 2014;74(3):198-200. Spanish. PMID: 24918666.
14. Malbrán A, Fernández Romero DS, Menéndez A. Angioedema hereditario. Guía de tratamiento [Hereditary angioedema. A therapeutic guide]. Medicina (B Aires). 2012;72(2):119-23. Spanish. PMID: 22522852.
15. Caballero T, Baeza ML, Cabañas R, Campos A, Cimbollek S, Gómez-Traseira C, González-Quevedo T, Guilarte M, Jurado-Palomo GJ, Larco JI, López-Serrano MC, López-Trascasa M, Marcos C, Muñoz-Caro JM, Pedrosa M, Prior N, Rubio M, Sala-Cunill A; Spanish Study Group on Bradykinin-Induced Angioedema; Grupo Español de Estudio del Angioedema mediado por Bradicinina. Consensus statement on the diagnosis, management, and treatment of angioedema mediated by bradykinin. Part I. Classification, epidemiology, pathophysiology, genetics, clinical symptoms, and diagnosis. J Investig Allergol Clin Immunol. 2011;21(5):333-47; quiz follow 347. Erratum in: J Investig Allergol Clin Immunol. 2012;22(2):3 p following 153. PMID: 21905496.
16. Caballero T, Baeza ML, Cabañas R, Campos A, Cimbollek S, Gómez-Traseira C, González-Quevedo T, Guilarte M, Jurado-Palomo J, Larco JI, López-Serrano MC, López-Trascasa M, Marcos C, Muñoz-Caro JM, Pedrosa M, Prior N, Rubio M, Sala-Cunill A; Spanish Study Group on Bradykinin-Induced Angioedema (SGBA). Consensus statement on the diagnosis, management, and treatment of angioedema mediated by bradykinin. Part II. Treatment, follow-up, and special situations. J Investig Allergol Clin Immunol. 2011;21(6):422-41; quiz 442-3. Erratum in: J Investig Allergol Clin Immunol. 2012;22(2):3 p following 153. PMID: 21995176.

**Unmet needs and other remarks:**

**^India:**

1. Lack of awareness about HAE and very few trained immunologists leading to remarkable delays in diagnosis.
2. Diagnostic facilities not available at most centres
3. Non-availability of all first line medications for acute treatment, short-term prophylaxis and long-term prophylaxis

**^^Italy:**

1. Delay of 12-24 months between approval of a new drug by the European Medicines Agency (EMA) and approval by AIFA (Italian government agency for drugs). Without this a new drug cannot enter the pharmacopoeia.
2. Possibility for some regions to impose more restrictive rules for the supply of drugs than those introduced by AIFA.
3. Drugs for HAE can only be prescribed in Italy by reference centres.
4. Patients can have the drugs at home and self-administration is allowed for all the therapies.
5. Acute treatment medications are available in most emergency rooms and are included in the National Poison Control Centre’s list of antidotes.

**^^Turkey:**

1. There are very few Allergy and Immunology specialists in the country
2. All patients need to contact Allergy specialists to get their insurance papers signed so that medications can be availed free of cost to them.
3. Patients with HAE who need prophylactic treatment with Cinryze need to apply to Allergy centres for completing “off-indication form” (Cinryze is not yet indicated for prophylaxis).
4. The most challenging problem would be the price of a new drug. At acceptable price levels, ministry asks HAE experts if new drug is required for patients

# Funded by National Health Service (NHS) only in some regions, whilst the three other treatments are funded by NHS everywhere

## Only for patients who need 4 or more OD treatments for acute attacks per month, who are intolerant or refractory to danazol or for whom danazol is contraindicated

### Only in some regions for Ruconest, everywhere for all the other above-mentioned drugs

**^^^Hungary:**

In Hungary, all HAE-related diagnoses and initiation of treatment are centrally managed by the National Angioedema Centre of Excellence and Reference, Semmelweis University, Budapest since 1990’s, and closely monitored in the National HAE Registry. Danazol has to be imported on an individual basis. The approved parenteral LTP medications are still under negotiation for reimbursement. While the National AE Center head is consulted for data supporting the actual needs, patient organizations have little if any influence on the reimbursement negotiation process. Price volume agreements place a cap on the reimbursement budget, and manufacturers have to cover any excess use. There is an especially burning unmet need of LTP for patients with frequent attack rates. The National AE Centre has to submit individual funding requests for approval by the National Health Insurance Fund Administration, which is a time-consuming administrative process for both the physician and the agency.

**^^^^North Macedonia**

LTP is an unmet need so far. There is a dynamic partnership between patients and health care professionsl in deciding upon new medications.

**^^^^^Spain**:

Although Berinert, Cinryze and icatibant are fully funded by National Health System, this is fully paid by hospital budget (funded by regional government) and the access to medication is very different throughout the country because of the high economic burden of these drugs for the hospital budget.

**^^^^^^^Bangladesh:**

1. Lack of awareness about HAE and very few trained immunologists leading to remarkable delays in diagnosis.
2. Diagnostic facilities not available at most centres
3. Non-availability of all first line medications for acute treatment, short-term prophylaxis and long-term prophylaxis

**Abbreviations:** HAE: hereditary angioedema; FFP: fresh frozen plasma; C1-INH: C1 inhibitor; USD: US dollar; TA: Tranexamic acid; iv: intravenous; sc: subcutaneous; STP: short term prophylaxis; LTP: long term prophylaxis; EAACI: European Academy of Allergy and Clinical Immunology; WAO: World Allergy Organization; pd: plasma derived: r: recombinant
